# Post-COVID-19 Pulmonary Inflammatory Pseudotumors Treated With Steroid Taper

**DOI:** 10.7759/cureus.57339

**Published:** 2024-03-31

**Authors:** Rayhan Karimi, Arun Adlakha

**Affiliations:** 1 Internal Medicine, Edward Via College of Osteopathic Medicine, Spartanburg, USA; 2 Pulmonology, Carolina Lung Clinic, Piedmont Medical Center, Rock Hill, USA

**Keywords:** cavitary lung disease, prednisone treatment, latent tuberculosis infection, corticosteroid treatment, pulmonary inflammatory pseudotumor, covid-19

## Abstract

The aftermath of COVID-19 continues to unveil an array of pulmonary complications, extending beyond the acute phase of the viral infection. Among these emerging sequelae, we present the case of a 58-year-old individual who developed pulmonary inflammatory pseudotumors (PIPs) following recovery from COVID-19. PIPs are exceedingly rare benign lesions that can pose a diagnostic challenge due to their clinical and radiological resemblance to malignant neoplasms. Histologically, PIPs are characterized by a proliferation of myofibroblastic spindle cells accompanied by inflammatory infiltrates, including lymphocytes, plasma cells, and histiocytes. As our understanding of post-COVID-19 complications evolves, this case serves as the first exploration into the complex interplay between COVID-19 infections and the subsequent development of inflammatory pseudotumors. In this report, an investigation is performed into the clinical presentation, diagnostic challenges, and successful management of post-COVID-19 PIPs with a focus on the pivotal role of corticosteroid therapy in mitigating the inflammatory response associated with this unique post-viral entity and resolution of the masses.

## Introduction

A pulmonary inflammatory pseudotumor (PIP) is a rare, benign lesion characterized by localized inflammatory tissue resembling a tumor in the lungs, often presenting with nonspecific respiratory symptoms. Histologically, PIPs are characterized by a proliferation of myofibroblastic spindle cells accompanied by inflammatory infiltrates, including lymphocytes, plasma cells, and histiocytes [1]. Out of all tumors located in the lung parenchyma and bronchus, PIPs represent about 0.7% [2].

Typically, first-line imaging includes X-ray and CT scans; however, these masses can exhibit heterogeneous characteristics on CT, ranging from solid to ground-glass opacities, making it challenging to differentiate them from malignancies based solely on radiographic findings. Additionally, inflammatory pseudotumors may lack distinctive features that unequivocally distinguish them from other pulmonary lesions. Positron emission tomography scans may provide additional information by assessing metabolic activity, but false-positive results are common and can further complicate the diagnostic process [3].

Furthermore, the diagnosis of PIP poses a significant dilemma for clinicians due to its rarity and the overlapping clinical and radiological features with malignant neoplasms or post-COVID-19 complications. The nonspecific nature of symptoms, such as persistent cough and dyspnea, coupled with radiographic findings, often prompts initial consideration of malignancy. Additionally, studies have shown that 30%-70% of patients have been shown to be asymptomatic, which adds another layer of complexity for diagnosis [4]. Distinguishing inflammatory pseudotumors from true malignancies remains challenging, requiring invasive procedures such as biopsy or surgical resection for definitive diagnosis [5].

Treatment options for PIPs are largely contingent upon the severity of symptoms, lesion size, and the presence of associated complications. In many cases, a conservative approach may be considered, involving close monitoring and serial imaging to observe the lesion's stability or regression over time. However, due to the potential for persistent symptoms and the risk of complications, therapeutic intervention may be warranted. Current treatment options include steroids, surgical resection, nonsteroidal anti-inflammatory drugs (NSAIDs), and radiotherapy [5]. Corticosteroids, such as prednisone or methylprednisolone, can be used as first-line treatments due to their conservative nature, demonstrating efficacy in mitigating the inflammatory response associated with PIPs. Surgical resection often serves as the definitive treatment option and has the lowest risk of recurrence; however, it is the most invasive. Surgical resection may be contemplated for cases with significant symptoms, lesions refractory to medical management, or when malignancy cannot be confidently ruled out.

The administration of steroids in patients with latent tuberculosis carries a substantial risk due to the potential for tuberculosis reactivation and the development of cavitary lung disease. Steroids, particularly high-dose or prolonged courses, can compromise the immune response, reactivating dormant *Mycobacterium tuberculosis* infections. Latent tuberculosis may remain clinically silent for years, and the immunosuppressive effects of steroids or COVID-19 can lead to uncontrolled bacterial replication. Reactivation can manifest as cavitary lung disease, a severe form of tuberculosis characterized by the formation of cavities within the lungs, which not only increases the risk of transmission but also escalates the morbidity and mortality associated with the disease [6]. Therefore, it is imperative for healthcare providers to thoroughly screen for latent tuberculosis before initiating steroid therapy, and if latent tuberculosis is identified, appropriate anti-tubercular prophylaxis or treatment should be instituted concurrently with steroid administration to mitigate the risk of reactivation and associated complications.

The diagnostic ambiguity underscores the importance of a multidisciplinary approach, incorporating clinical, radiological, and pathological assessments, to ensure accurate identification and subsequent tailored management strategies for patients with post-COVID-19 PIPs. This case represents the first documented case of post-COVID-19 PIPs, displaying the need for additional investigation into their presentation, diagnosis, and therapeutic options.

## Case presentation

**Figure 1:**
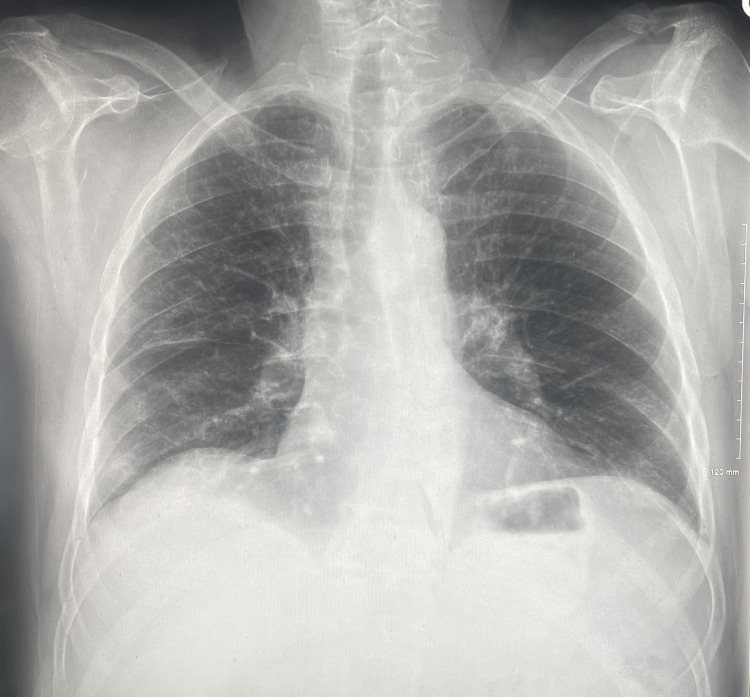
Initial chest X-ray in the ER displaying bilateral, peripheral, patchy airspace opacities.

We present the case of a 58-year-old male with a history of chronic obstructive pulmonary disease (COPD), emphysema, a 40-pack-year smoking history, and anxiety, presenting to the ER exhibiting a seven-day history of progressive cough, shortness of breath, vomiting, diarrhea, and a low-grade fever. Clinical examination in the ER revealed a pulse oxygen of 89%, tachycardia, and a blood pressure of 148/72. Laboratory tests showed leukocytosis of 16,000 with mild neutrophilia and lymphopenia, which was concerning for a COPD flare-up. A chest X-ray was completed, shown in Figure 1, revealing bilateral, peripheral, patchy airspace opacities.

These findings resembled multifocal pneumonia; therefore, respiratory syncytial virus (RSV), influenza, and COVID-19 tests were performed. The COVID-19 test came back strongly positive. He was placed on oxygen, IV dexamethasone, remdesivir, and oral doxycycline. He responded well to this treatment and was weaned off the oxygen over the course of the hospital stay. Another chest X-ray was performed prior to discharge, which displayed improved bilateral lung infiltrates. The patient was then discharged home in stable condition.

**Figure 2:**
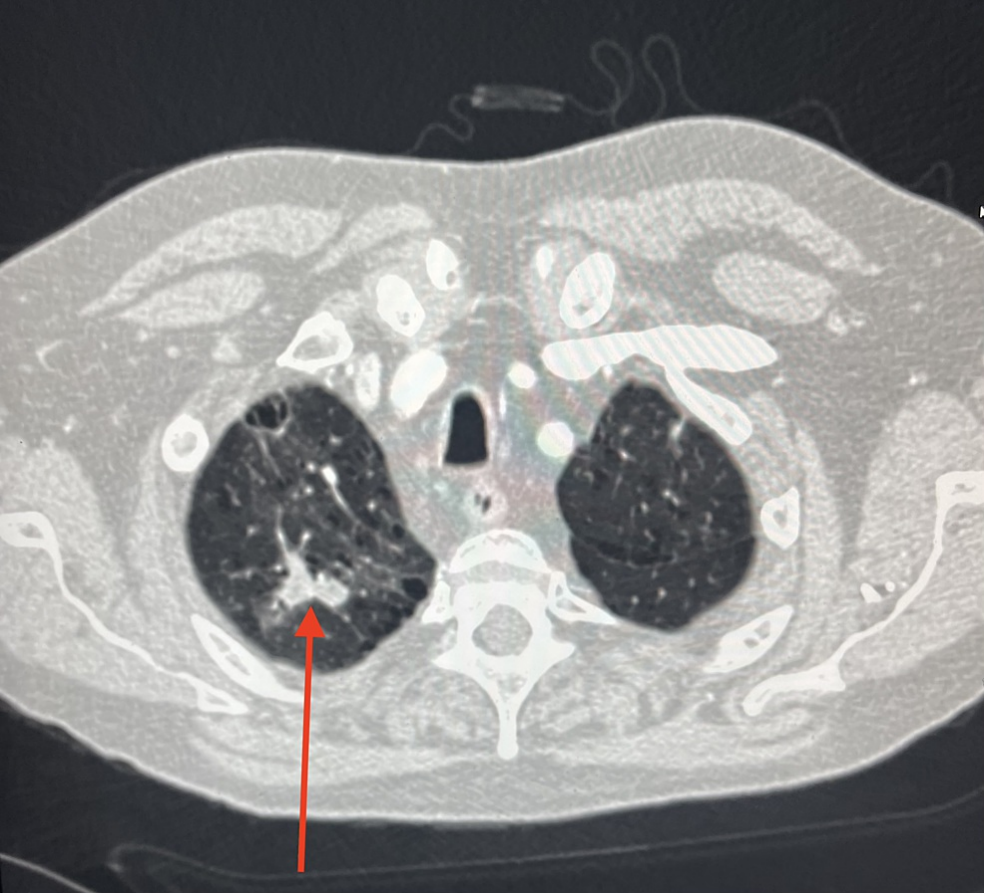
CTA of the chest displaying a 3×2 cm spiculated consolidation in the right upper lung lobe. CTA: CT angiography

**Figure 3:**
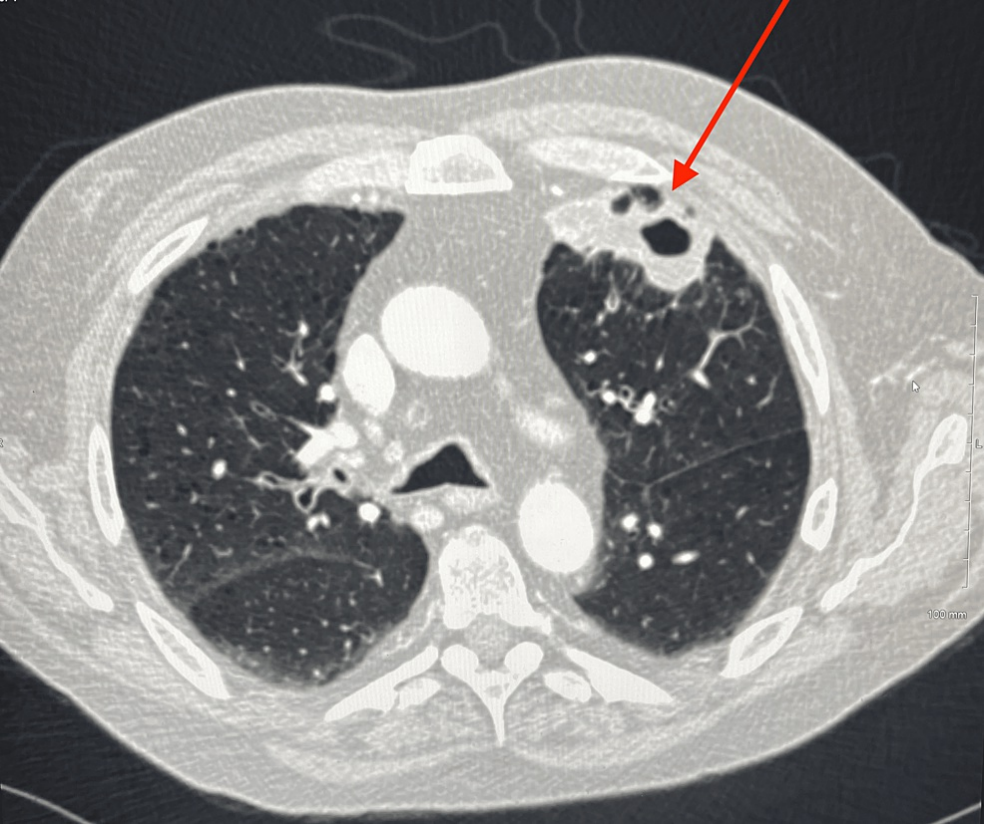
CTA of the chest showing a 6×3×6 cm partially cystic focal consolidation in the anterior left lobe. CTA: CT angiography

The patient remained clinically stable until the following summer when he noted increasing mucoid productive sputum, cough, and shortness of breath. A repeat chest X-ray showed a cavitary lesion in the left upper lobe with a possible air-fluid level. Additionally, a CT angiography (CTA) of the chest was performed, and a new right upper lobe spiculated consolidation was noted (Figure 2) along with left cystic consolidations in the left anterior lobe (Figure 3).

**Figure 4:**
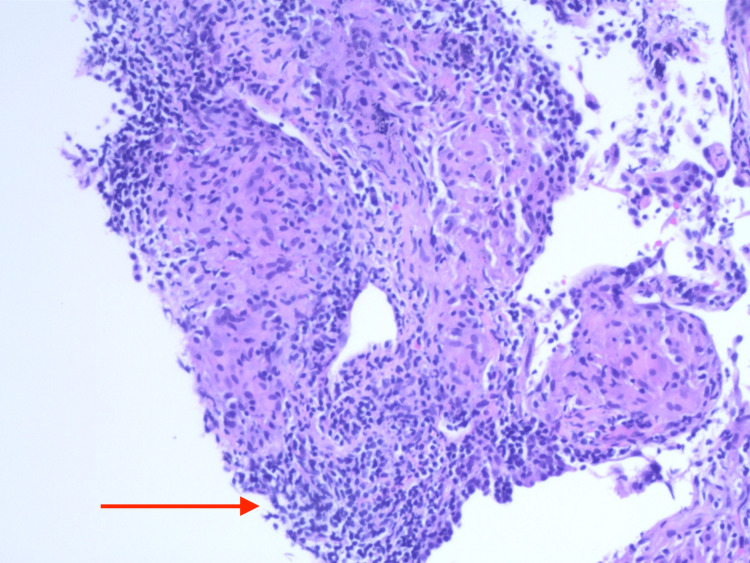
Histology slide from lung biopsy showing a non-caseating granuloma with interspersed giant cells and background chronic inflammation.

**Figure 5:**
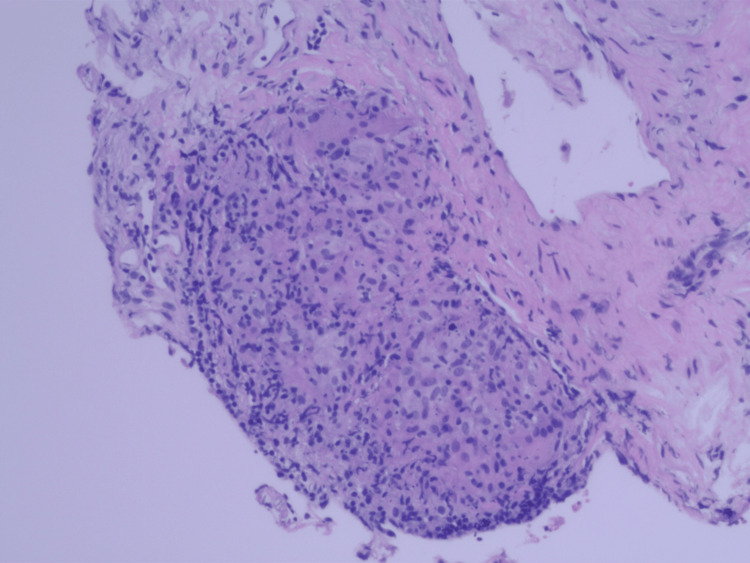
Histology slide from lung biopsy showing a poorly formed granuloma comprised of histiocytes and lymphocytes.

In light of the CTA of the chest findings of new right upper lobe and left anterior lobe lesions, a CT-guided right upper lobe lung biopsy was performed and sent off for histopathology as well as microbiology evaluation. Results from touch prep showed histiocytic aggregates and other features consistent with granulomatous inflammation (Figure 4, Figure 5). The core biopsy confirmed focally necrotizing granulomatous inflammation and showed no evidence of malignancy or vasculitis.

**Figure 6:**
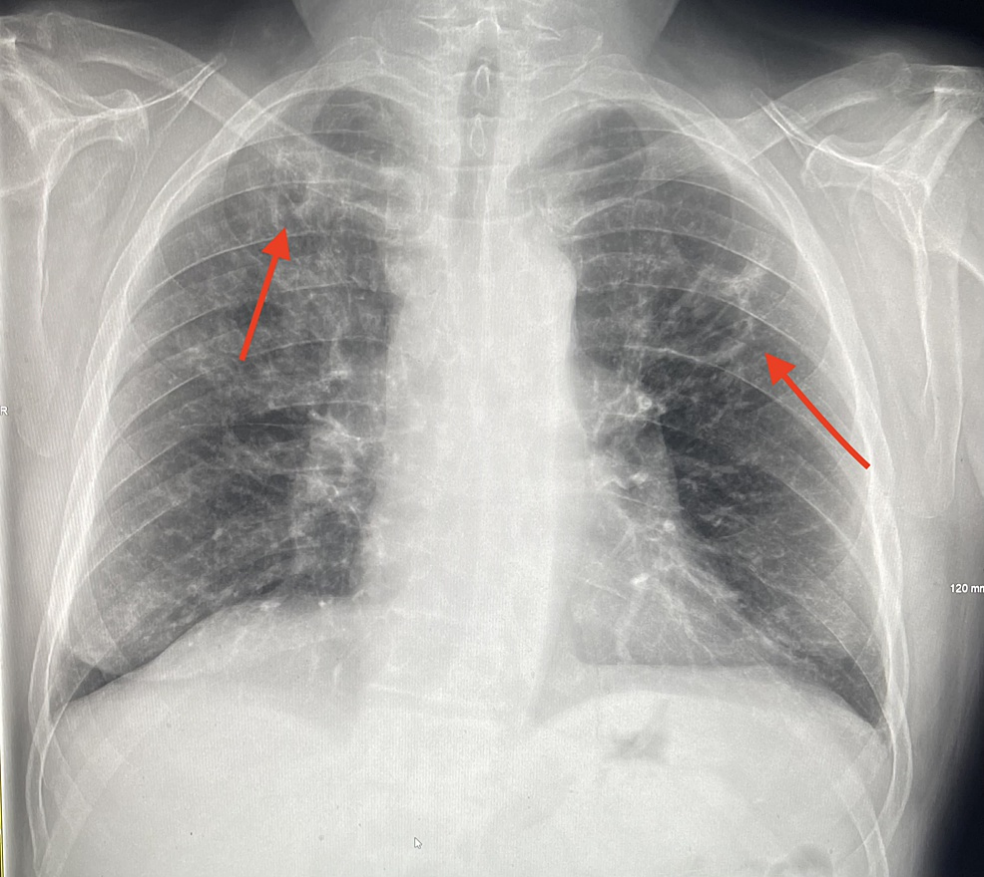
Chest X-ray post-steroid taper showing residual areas of linear opacification of the lungs, within lung apices, suggesting areas of scarring.

All of the special stains were negative for acid-fast bacilli (AFB) and fungal elements. In consideration of the symptomatic and inflammatory nature of the bilateral upper lung mass-like lesions, the patient was placed on a slow prednisone taper, starting at 0.75 mg/kg, which continued over the course of six months. Chest X-ray (post-steroids) showed a marked decrease in the size of the masses with signs of scarring, as seen in Figure 6. The patient did well on the steroids and returned back to baseline health.

**Figure 7:**
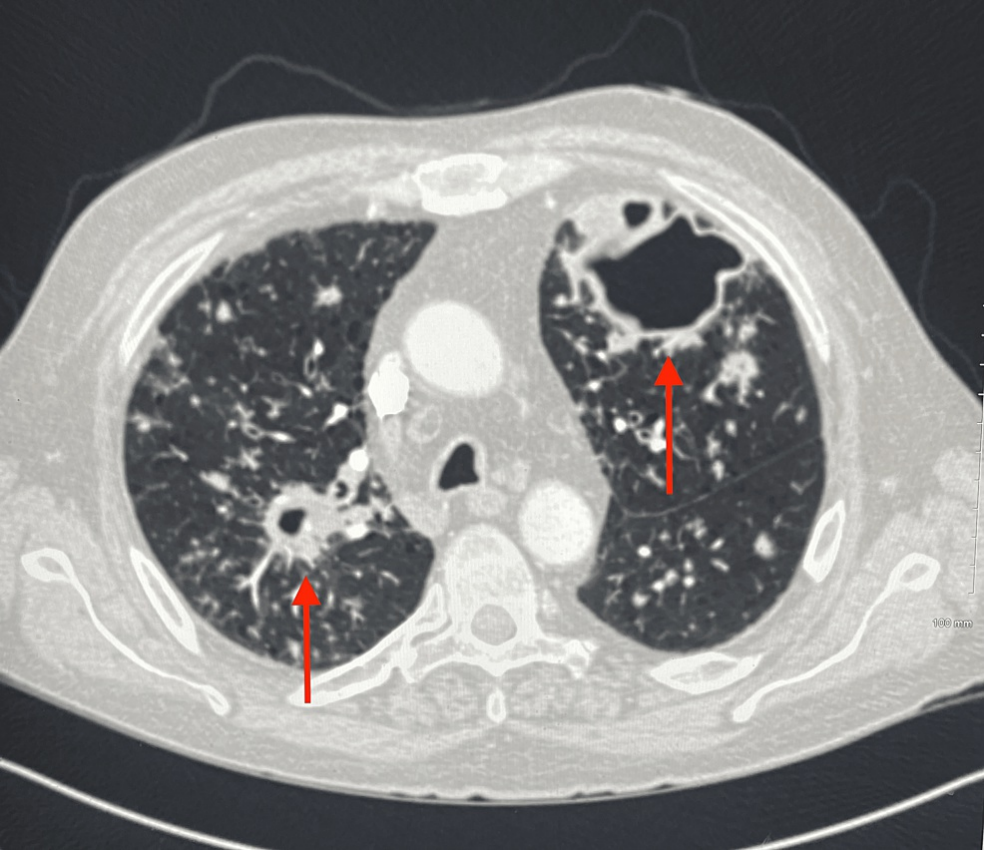
CTA of the chest showing progressive bilateral cavitary disease.

The following spring, the patient developed increasing cough, dyspnea, night sweats, low-grade fever, anorexia, and weight loss, prompting further investigation. Lab work-up revealed elevated erythrocyte sedimentation rate (ESR), C-reactive protein (CRP), leukocytosis, and anemia. Another CTA of the chest was performed, which showed extensive bilateral cavitary lung disease (Figure 7).

Pulmonary involvement was noted to be fairly diffuse. Multiple sputum AFB smears revealed the presence of >50 to >200 AFB per field with the final identification of the sputum AFB cultures being *M. tuberculosis*. The patient was initiated on daily observed therapy with isoniazid, rifampin, ethambutol, pyrazinamide, and vitamin B6. He tolerated the treatment very well without any significant side effects of the drug regimen. He noted improvement in both respiratory and constitutional symptoms and was once again returning to baseline health. 

## Discussion

The treatment of PIPs is multifaceted and depends on the severity of symptoms, lesion size, and potential complications. Given the rarity of PIPs and their resemblance to malignant neoplasms, a definitive diagnosis is crucial for appropriate management. Therefore, after identification through radiology, a biopsy is needed to rule out malignancy, which is imperative to determine precise treatment for patients and remains the gold standard for diagnosis. In our case, a CT-guided lung biopsy was performed which yielded adequate results for the determination of PIPs. 

The first treatment option that can be implemented is watchful waiting with serial X-rays and CT scans. However, this option has the inherent risk of mass growth and compression of nearby structures. PIPs can be highly aggressive and invade the bronchi, mediastinum, or chest wall, requiring the next treatment option, which is the most definitive and invasive, surgical resection [7]. Resection allows for complete histological examination of the masses and also has the lowest rate of recurrence. Studies have shown that the prognosis for surgical resection is excellent [8]. However, to mitigate the risk of recurrence, surgeons often suggest segmentectomy or lobectomy, which is a highly invasive procedure and can be frightening for patients. In contrast, another treatment option is the use of corticosteroids, which was implemented in our case. In a review of the medical literature, steroids have been shown to be used in patients when the mass is too close to a structure or the patient is not a surgical candidate. Our case and two other reported cases of PIPs were successfully treated with a prednisone taper [9]. In another case report of a patient who had a recurrence of masses after surgical resection, a prednisolone taper was used and successfully resolved the masses within 45 days of treatment [10]. Furthermore, in a separate case series, three patients who failed surgical resection were subsequently treated with steroids, which resulted in the resolution of the masses with no relapse after discontinuation of the steroids [11]. Therefore, with our presented case in conjunction with the current medical literature, it can be posed that corticosteroids are efficacious in the treatment of PIPs.

In recommendation to practicing clinicians who encounter patients with post-COVID-19 PIPs, it is proposed that a steroid taper can be used as first-line therapy due to its minimal invasiveness and proven successful treatment. Additionally, serial imaging is indicated to assess for recurrence or metastases. 

In review, this case presents a distinctive aspect in the medical literature, marking it as the inaugural documented instance of PIPs occurring subsequent to COVID-19 infection. This unprecedented association highlights the expanding spectrum of potential post-viral sequelae related to COVID-19, shedding light on the intricate interplay between viral infections and the development of inflammatory pseudotumors.

Moreover, it was noted that after the patient sustained a COVID-19 infection along with steroid immunosuppression in the treatment of the PIPs, his latent tuberculosis infection was reactivated. Therefore, patients who have post-COVID-19 PIPs and who are candidates for a steroid course should undergo a QuantiFERON blood test to rule out latent tuberculosis before initiation. This will mitigate the risk of cavitary disease as seen in this case. 

## Conclusions

This case represents a pivotal contribution to the medical literature as the first documented occurrence of PIPs following a COVID-19 infection. The successful management of this unique case, involving a carefully administered steroid taper, underscores the significance of tailored treatment approaches in addressing the inflammatory nature of PIPs. Additionally, the concomitant identification of tuberculosis infection during the course of this case serves as a reminder of the importance of remaining vigilant in the post-COVID-19 landscape. This displays the necessity for comprehensive patient evaluations when using steroids, considering latent infections such as tuberculosis, especially in those with persistent respiratory symptoms. One limitation of this case is the inability for long-term follow-up. Although the patient has not had a recurrence of the masses, that always remains a possibility and can happen in the future. Therefore, additional research should be performed to assess steroid efficacy long-term in PIPs. The multifaceted nature of this case highlights the evolving setting of post-COVID-19 complications and emphasizes the critical role of continuous research and vigilant clinical care in understanding and addressing the diverse sequelae associated with this pandemic.
